# Aggressive fluid accumulation is associated with acute kidney injury and mortality in a cohort of patients with severe pneumonia caused by influenza A H1N1 virus

**DOI:** 10.1371/journal.pone.0192592

**Published:** 2018-02-15

**Authors:** Gustavo Alejandro Casas-Aparicio, Isabel León-Rodríguez, Rafael de Jesús Hernández-Zenteno, Manuel Castillejos-López, Claudia Alvarado-de la Barrera, Christopher E. Ormsby, Gustavo Reyes-Terán

**Affiliations:** 1 Centro de Investigación en Enfermedades Infecciosas, Instituto Nacional de Enfermedades Respiratorias Ismael Cosío Villegas, Ciudad de México, México; 2 Servicio Clínico 5, Instituto Nacional de Enfermedades Respiratorias Ismael Cosío Villegas, Ciudad de México, México; 3 Departamento de Epidemiología, Instituto Nacional de Enfermedades Respiratorias Ismael Cosío Villegas, Ciudad de México, México; University of Sao Paulo Medical School, BRAZIL

## Abstract

**Introduction:**

Fluid accumulation is associated with adverse outcomes such as acute kidney injury (AKI) in critically ill patients. This study aimed to describe the factors associated with AKI in individuals with influenza A H1N1 severe pneumonia, and explore the relation of fluid accumulation with AKI and mortality.

**Material and methods:**

We reviewed medical records of individuals with influenza A H1N1 severe pneumonia and no history of chronic kidney disease, attending a national referral center for respiratory diseases between November 2014 and May 2015. Demographic information, risk factors for AKI, physiologic and laboratory data, outcomes and information on fluid intake and output were recorded. Categorical variables were compared using the chi-square test. Quantitative variables were compared using the Mann-Whitney test. Factors associated with AKI and mortality were identified by binary logistic regression. Linear models of fluid accumulation rates for individuals and groups were estimated using segmented linear regression.

**Results:**

Of 60 patients studied, 43 developed AKI (71.6%). Male gender was protective for AKI (p = 0.019). AKI was associated with nephrotoxic drugs (p = 0.016); PEEP>10 cm H_2_O on admission (p = 0.031); mortality (p = 0.037); and fluid accumulation ≥10% (fluid overload) at day 7 of hospitalization (p = 0.00026). Mortality was associated with older age (p = 0.009); nephrotoxic drugs (p = 0.034); and higher Pneumonia Severity Index score (112 vs. 76, p = 0.008) on admission. The Deceased-AKI group had a higher rate of fluid accumulation (expressed as ml/kg/body weight) than the Survivors-No AKI group during the study period of 7 days (Survivors-No AKI = 13.31 vs. Deceased-AKI = 22.76, p = 0.019). During the highest phase of fluid accumulation, the Survivors-No AKI group had a slower rate of fluid accumulation than the Survivors-AKI group (14.91 vs. 28.49, p = 0.001).

**Conclusions:**

A high rate of fluid accumulation was associated with AKI and mortality. We support the approach of resuscitation in acute illness, with an early transition to neutral and then negative fluid balances.

## Introduction

Influenza A H1N1 virus infection is associated with a spectrum of illnesses, ranging from upper respiratory infection, to multiple organ dysfunction and death. In critical patients with severe influenza A H1N1 disease, acute kidney injury (AKI) is a common complication. AKI is associated with development of chronic kidney disease [1], increased mortality, adverse outcomes and longer periods of intensive care unit stay [2, 3]. In most cases, AKI develops from a combination of factors including hypovolemia, sepsis, nephrotoxins and hemodynamic perturbations [4]. In order to restore cardiac output, systemic blood pressure and renal perfusion in critically ill patients, an adequate fluid resuscitation is necessary [5]. However, fluid administration beyond the correction of hypovolemia is associated with AKI, longer periods of hospital stay, increased mortality [6], organ dysfunction and worse clinical outcomes [7]. Fluid overload (FO) may derive from the combination of oliguria and fluid administration, leading to a positive fluid balance [8, 9]. Due to the dichotomy between traditional teaching and evolving evidence, wide variations in clinical fluid management exist and traditional practice involving administration of large fluid volumes is being questioned [5]. Prolonged fluid resuscitation leads to edema in the kidneys and other organs. As an encapsulated organ, the kidney is particularly affected by fluid congestion and raised venous pressures with a disproportionate elevation of intracapsular pressure, which leads to a decrease in renal blood flow and glomerular filtration rate [10].

The association between FO and AKI has been consistently reported, but the cause-effect relationship remains unclear. The aim of this study was to describe the factors associated with the development of AKI in critically ill individuals with influenza A H1N1 severe pneumonia, and we had a particular interest in exploring the relation of fluid accumulation on the development of AKI and mortality.

## Material and methods

### Study population

This study was conducted at the National Institute of Respiratory Diseases (INER), a national referral center in Mexico City. We retrospectively reviewed the medical records of individuals with severe pneumonia caused by influenza A H1N1 attending our institution between November 2014 and May 2015. Patients with influenza A H1N1 severe pneumonia were those with clinical data of respiratory distress, bilateral alveolar opacities in 2 or more lobes, a ratio of PaO_2_/FiO_2_ < 200 mm Hg, and a positive result for the influenza A H1N1-rRT-PCR assay in nasopharyngeal swab or bronchoalveolar lavage samples.

### Procedures

The retrospective review of medical records included demographic and anthropometric variables, comorbidities, clinical and laboratory data, initiation and termination dates of mechanical ventilation, days on intensive care unit (ICU), initial mechanical-ventilator settings and use of vancomycin or other nephrotoxic drugs. The Acute Physiology and Chronic Health Evaluation (APACHE) II score, as well as the Sequential Organ Failure Assessment (SOFA) and the Pneumonia Severity Index (PSI) scores were calculated on admission. Information on fluid intake and output was obtained from fluid balance monitoring charts. Daily fluid balance was determined from all intakes and outputs recorded. All available intake and output data from 7 days in hospital were included in the analyses. We computed fluid balance for each day using the sum of daily fluid intake (L) from which we subtracted total output (L). To quantify cumulative fluid balance in relation to body weight, we used the following formula: (∑ daily (fluid intake (L)–total output (L)) / body weight (in kilograms). We used the term ‘percentage of fluid accumulation’ to define the percentage of cumulative fluid balance adjusted for body weight. Baseline body weight was based on initial hospital admission weight [11]. Total output included urinary volume, evacuations and insensible losses.

The relation of fluid accumulation with AKI and mortality was explored by comparing the frequency of these outcomes in individuals with fluid accumulation <10% versus those with fluid accumulation ≥10% (FO).

### Inclusion and exclusion criteria

We included individuals with diagnosis of severe pneumonia caused by influenza A H1N1, confirmed by real-time reverse transcription–polymerase chain reaction (rRT-PCR); age 18 or older; estimated glomerular filtration rate (eGFR) > 60 ml/min/1.73 m^2^ on admission using the Chronic Kidney Disease Epidemiology Collaboration (CKD-EPI) equation; no history of chronic kidney disease (CKD); and ratio of partial pressure arterial oxygen/fraction of inspired oxygen (PaO_2_/FiO_2_) <200 mm Hg on admission. Pregnant women were not included in the study. The Research and Ethics Committee of the INER approved the study and waived the requirement for informed consent due to the retrospective design of the study.

### Definitions

Fluid overload was defined as fluid accumulation greater than or equal to 10% of the baseline body weight [12]. Serum creatinine (sCr) was adjusted for fluid accumulation at the moment of AKI diagnosis [13]. Adjusted sCr (sCr′) was calculated as:

$$sCr^{\prime }=sCr[1+(\frac{Cumulative\;FB}{0.6BW})]$$

Where FB is fluid balance and 0.6BW is 60% of body weight in kg, equivalent to total body water.

Diagnosis of AKI was based on a rapid reduction of kidney function, defined by an absolute increase in adjusted sCr >0.3 mg/dL within 48 hours compared to baseline; a sCr increase of 1.5 times within 7 days compared to baseline; or a decrease in urinary output >0.5 ml/kg/hour for more than six hours. AKI stage was defined according to the Kidney Disease Improving Global Outcomes (KDIGO) staging system [14]. AKI stage 1 corresponded to a sCr increase of 1.5–2 times baseline; AKI stage 2 corresponded to a sCr increase 2.0–3.0 times baseline; and AKI stage 3 corresponded to a sCr increase of >3 times baseline or initiation of renal replacement therapy (RRT). Information about administration of nephrotoxic drugs before AKI was also retrieved from clinical files. Nephrotoxic drugs were defined as those causing tubular or renal hemodynamic toxicity, and included nonsteroidal anti-inflammatory drugs, aminoglycosides, vancomycin, colistine and amphotericin B.

### Statistical analysis

Categorical variables were compared using the chi-square test. Median and interquartile ranges (IQRs) were calculated for quantitative variables and differences between groups were analyzed using the Mann-Whitney test. Odds ratios (ORs) and 95% confidence intervals (CIs) were obtained using logistic regression analyses. A two-sided P value <0.05 was considered to be significant. Statistical analyses were performed using SPSS version 20 (SPSS Inc., Chicago, IL).

We explored if a fluid accumulation ≥10% body weight gain was a predictor of AKI or death. Then we analyzed if the rate of fluid balance increase, indicated by the slope of the relation of body weight gain/day, was a predictor of AKI or mortality. This analysis was performed considering total volume or volume per kg of body weight. We estimated individual linear models having one or more segmented relationships in the linear predictor. Individuals with no converging segments were assumed to have a single segment across the follow-up period of 7 days. Estimates of the slopes and the possibly multiple breakpoints for each patient were obtained by segmented linear regression, using Package segmented version 0.5–1.4 for the R statistical language, Muggeo V.M.R. [15].

## Results

During the period between November 2014 and May 2015, 184 individuals were diagnosed with severe pneumonia at our institution. Of those, 124 were deemed ineligible for this study because they did not fulfill the inclusion criteria (8 were under 18 years of age; 22 had incomplete clinical files; 9 had non-H1N1 pneumonia; and on the remaining 85 individuals, either influenza A H1N1 could not be confirmed by rRT-PCR, or they had PaO_2_/FiO_2_ >200 mm Hg). We thus included 60 individuals in the study. Of those, 39 were men (65%). The median age was 47.5 years, IQR, 44.2–50.9; 56.7% were obese; 21.7% had systemic arterial hypertension; 8.3% had diabetes mellitus; 6.7% had pneumopathies; and 38.3% had a smoking history. Only 1.7% of this cohort had been vaccinated against influenza. Sudden onset of influenza symptoms (<72 hours) was observed in 63.3% of the cases. The median APACHE II score on admission was 13.5 (IQR, 9.25–17). Forty-three individuals developed AKI (the AKI group). Of those, 9 had AKI stage 1 (20.8%); 16 had AKI stage 2 (37.1%) and 18 had AKI stage 3 (41.7%). Four patients requiring replacement therapy (6.7%) received sustained low-efficiency dialysis, and all of them survived.

### Factors associated with acute kidney injury

Demographic and clinical characteristics of the AKI group (n = 43) vs. the non-AKI group (n = 17) on admission are shown in Table 1.

**Table 1:** Characteristics of the Acute Kidney Injury Group and the non-Acute Kidney Injury Group on admission.

Variables	AKI, n = 43 Median (% or IQR)	Non-AKI, n = 17 Median (% or IQR)	P
Male	24 (55.8%)	15 (88.2%)	0.019
Diabetes	5 (11.6%)	0 (0%)	0.309
Hypertension	12 (27.9%)	1 (5.9%)	0.086
Obesity	27 (62.8%)	7 (41.2%)	0.128
Smoking	29 (67.4%)	8 (47.1%)	0.143
Fever before admission	41 (95.3%)	13 (76.5%)	0.048
Antibiotics before admission	37 (86.0%)	12 (70.6%)	0.265
Nephrotoxic drugs	25 (58.1%)	4 (23.5%)	0.016
PEEP level >10 (cm H_2_O)	15 (34.8%)	10 (5.9%)	0.031
Deceased	17 (39.5%)	2 (11.8%)	0.037
Age (years)	45 (IQR, 38–52)	49 (IQR, 39–56)	0.431
PSI score	81 (IQR, 64–114)	76 (IQR, 64–105)	0.774
APACHE II score	14 (IQR, 10–18)	12 (IQR, 8–16)	0.243
SOFA score	7 (IQR, 4–10)	5 (IQR, 3.5–9)	0.204
pH	7.39 (IQR, 7.34–7.46)	7.42 (IQR, 7.39–7.46)	0.650
PaO_2_ (mmHg)	39 (IQR, 31–52)	35 (IQR, 28–42)	0.325
pCO_2_ (mmHg)	33 (IQR, 28–50)	33 (IQR, 27–40)	0.935
HCO_3_ (mmol/L)	22 (IQR, 18–26)	25 (IQR, 22–26)	0.118
PaO_2_/FIO_2_ (mmHg)	93 (IQR, 61–132)	118 (IQR, 62–154)	0.333
Hemoglobin (g/dL)	15 (IQR, 14–17)	16 (IQR, 14–16.5)	0.659
Leucocytes (x10^9^/L)	7400 (IQR, 5800–11300)	7900 (IQR, 5200–12600)	0.799
Platelets (x10^9^/L)	170 (IQR, 142–232)	199 (IQR, 136.5–265.5)	0.827
Urea nitrogen (mg/dL)	20 (IQR, 12–29)	16 (IQR, 12–24.6)	0.275
Creatinine (mg/dL)	1.0 (IQR, 0.95–1.73)	1.0 (IQR, 0.97–1.46)	0.264
Albumin (g/L)	2.74 (IQR, 2.1–3.0)	2.88 (IQR, 2.64–3.25)	0.272
LDH (U/L)	642 (IQR, 358–1091)	567 (IQR, 325–769)	0.827
CPK (U/L)	205 (IQR, 113–387)	329 (IQR, 120–1719)	0.513
AST (U/L)	73 (IQR, 40–120)	74 (IQR, 49.5–108)	0.827
Procalcitonin ng/mL	1.0 (IQR, 0.07.2.79)	0.38 (IQR, 0.05–1.89)	0.275
C-Reactive Protein (mg/L)	13.1 (IQR, 8.4–24.2)	2.97 (IQR, 0.78–4.1)	0.127
Glucose (mg/dL)	150 (IQR, 111–190)	129 (IQR, 99.5–153.5)	0.250
CKD-EPI (mL/min/1.73 m^2^ bs)	70 (IQR, 46.5–98)	86 (IQR, 58–102)	0.268
Cockcroft-Gault (mL/min)	90 (IQR, 55.40–120)	98 (IQR, 73–113)	0.724
Hospitalization (days)	29 (IQR, 14–50)	20 (IQR, 12–27.5)	0.081
Mechanical ventilation (days)	19.5 (IQR, 9.5–37.2)	10 (IQR, 5.5–26)	0.253

AKI, Acute kidney injury; IQR, Interquartile range; %, Percentage; PEEP, Positive end-expiratory pressure; PSI, Pneumonia Severity Index; APACHE II, Acute Physiology and Chronic Health Evaluation II; SOFA, Sequential Organ Failure Assessment; PaO_2_/FiO_2_, Pressure arterial oxygen/Fraction of inspired oxygen; LDH, Lactic dehydrogenase; CPK, Creatine phosphokinase; AST, Aspartate aminotransferase; CKD-EPI, Chronic Kidney Disease Epidemiology Collaboration; CKD-EPI is expressed in mL/min/1.73m^2^ body size.

Male gender was protective for AKI (24 males in the AKI group vs. 15 in the non-AKI group, p = 0.019, odds ratio (OR) = 0.16, CI, 0.03–0.82). AKI was associated with the use of nephrotoxic drugs (25 individuals in the AKI group vs. 4 in the non-AKI group, p = 0.016, OR = 4.51, CI, 1.26–16); and positive end-expiratory pressure (PEEP) level >10 cm H_2_O (15 individuals in the AKI group vs. 10 in individuals the non-AKI group, p = 0.031, OR = 5, CI, 1.09–22.82). Mortality was significantly more frequent in the AKI group (17 individuals in the AKI group vs. 2 individuals in the non-AKI group, p = 0.037, OR = 4.9, CI, 0.99–24.2). The AKI group had higher PSI, APACHE II and SOFA scores on admission, as well as longer hospital stays and mechanical ventilation periods than the non-AKI group, but these differences were non-significant. Laboratory values and gasometric variables on admission were not significantly different between both groups.

### Factors associated with mortality

Demographic and clinical characteristics of deceased individuals and survivors are shown in Table 2.

**Table 2:** Characteristics of deceased individuals and survivors on admission.

Variables	Deceased, n = 19 Median (% or IQR)	Survivors, n = 41 Median (% or IQR)	P
Male	11 (57.9%)	28 (68.3%)	0.432
Diabetes	3 (15.8%)	2 (4.9%)	0.314
Hypertension	6 (31.6%)	7 (17.0%)	0.312
Obesity	10 (52.6%)	24 (58.5%)	0.668
Smoking	5 (26.3%)	18 (43.9%)	0.192
Fever before admission	17 (89.5%)	37 (90.2%)	1.000
Antibiotics before admission	17 (89.5%)	32 (78.0%)	0.476
Nephrotoxic drugs	13 (68.4%)	16 (39.0%)	0.034
PEEP level >10 (cm H_2_O)	12 (63.1%)	5 (12.2%)	0.575
Age (years)	63 (IQR, 54–76)	54 (IQR, 43–60)	0.009
PSI score	112 (IQR, 72–128)	76 (IQR, 62.5–91.5)	0.008
APACHE II score	16 (IQR, 14.1–18.2)	12.4 (IQR, 10.9–13.9)	0.004
SOFA score	8 (IQR, 7–10.8)	6.05 (IQR, 5.14–6.96)	0.006
pH	7.38 (IQR, 7.30–7.46)	7.43 (IQR, 7.41–7.45)	0.780
PaO_2_ (mmHg)	34 (IQR, 29.0–40)	35.8 (IQR, 32.8–38.7)	0.046
pCO_2_ (mmHg)	34 (IQR, 29.0–40)	35.8 (IQR, 32.8–38.7)	0.184
HCO_3_ (mmol/L)	20 (IQR, 16.3–24.5)	24 (IQR, 19.5–26.35)	0.055
PaO_2_/FIO_2_ (mmHg)	125 (IQR, 99.6–150.5)	140 (IQR, 126.3–152.3)	0.361
Hemoglobin (g/dL)	15 (IQR, 14–16.3)	14.96 (IQR, 14–15.6)	0.595
Leucocytes (x10^9^/L)	8621 (IQR, 5975–11267)	9534 (IQR, 8017–11051)	0.361
Platelets (x10^9^/L)	163 (IQR, 116–224)	188 (IQR, 147–253)	0.127
Urea nitrogen (mg/dL)	27 (IQR, 17–38.16)	21 (IQR, 17.4–24.5)	0.633
Creatinine (mg/dL)	1.30 (IQR, 0.85–1.74)	1.30 (IQR, 1.1–1.49)	0.401
Albumin (g/L)	2.58 (IQR, 2.38–2.79)	2.84 (IQR, 2.66–3.01)	0.077
LDH (U/L)	770 (IQR, 373–1147)	583 (IQR, 352–767)	0.236
CPK (U/L)	217 (IQR, 132–918)	211 (IQR, 115–490)	0.751
AST (U/L)	82 (IQR, 41–134)	73.5 (IQR, 46.5–105.7)	0.703
Procalcitonin ng/mL	2.88 (IQR, 0.32–6)	1.85 (IQR, 0.56–3.13)	0.604
C-Reactive Protein (mg/L)	12.2 (IQR, 10.3–13.5)	8.9 (IQR, 3.97–28.1)	0.864
Glucose (mg/dL)	182 (IQR, 142–223)	144 (IQR, 126–160)	0.053
CKD-EPI (mL/min/1.73m^2^ bs)	71 (IQR, 42–90)	75 (IQR, 50–102.5)	0.499
Cockcroft-Gault (mL/min)	93 (IQR, 66–121)	97.4 (IQR, 82.2–112.6)	0.583
Hospitalization (days)	23 (IQR, 12–41)	27 (IQR, 14–42)	0.399
Mechanical ventilation (days)	23 (IQR, 8–41)	16.5 (IQR, 6.75–31.5)	0.971

AKI, Acute kidney injury; IQR, Interquartile range; %, Percentage; PEEP, Positive end-expiratory pressure; PSI, Pneumonia Severity Index; APACHE II, Acute Physiology and Chronic Health Evaluation II; SOFA, Sequential Organ Failure Assessment; PaO_2_/FiO_2_, Pressure arterial oxygen/Fraction of inspired oxygen; LDH, Lactic dehydrogenase; CPK, Creatine phosphokinase; AST, Aspartate aminotransferase; CKD-EPI, Chronic Kidney Disease Epidemiology Collaboration; CKD-EPI is expressed in mL/min/1.73m^2^ body size.

Older age was significantly associated with mortality (median age of 63 years, IQR, 54–76 in deceased individuals vs. 54 years, IQR, 43–60 in survivors, p = 0.009). The use of nephrotoxic drugs during hospitalization was significantly associated with mortality (45% in deceased individuals vs. 55% in survivors, p = 0.034). The Pneumonia Severity Index on admission was significantly higher in deceased patients than in survivors (PSI 112, IQR, 72–128 in deceased individuals vs. 76, IQR, 62.5–91.5 in survivors, p = 0.008). PaO_2_/FiO_2_ and HCO_3_ levels on admission were lower in deceased individuals than in survivors, but differences were non-significant.

### Relation of fluid accumulation with AKI and mortality

Cumulative fluid balance until day 7 of hospitalization was obtained for 53 individuals. We explored the relation of FO with AKI and mortality in this group. Fluid overload was associated with AKI (p = 0.00026, OR = 4.28). Mortality was more frequent in the group with FO, but the association was non-significant (p = 0.508, OR = 2.08).

In the group of 53 individuals, we also assessed the accumulated fluid balance per day (expressed in mL/Kg of body weight) during 7 days of hospitalization (Table 3). The accumulated fluid balance was higher in the group with AKI than in the non-AKI group on Day 3 (p = 0.001), Day 4 (p = 0.004), Day 5 (p = 0.018) and Day 6 (p = 0.023). The accumulated fluid balance was higher in the Deceased group than in Survivors on Day 7 (p = 0.038).

**Table 3:** Accumulated fluid balance per day during hospitalization.

	Day1 (mL/Kg)	Day 2 (mL/Kg)	Day 3 (mL/Kg)	Day 4 (mL/Kg)	Day 5 (mL/Kg)	Day 6 (mL/Kg)	Day 7 (mL/Kg)
AKI	31 (14–39)	52 (37–76)	84 (68–116)	116 (86–135)	128 (94–170)	135 (95–180)	143 (112–189)
No-AKI	19 (10–35)	42 (24–57)	53 (37–73)	78 (32–100)	70 (38–121)	84 (41–145)	116 (57–160)
AKI vs. No-AKI	P = 0.209	P = 0.086	P = 0.001	P = 0.004	P = 0.018	P = 0.023	P = 0.131
Deceased	32 (17–49)	56 (37–87)	90 (58–118)	122 (74–139)	147 (106–170)	160 (120–192)	178 (116–210)
Survivors	24 (11–37)	48 (34–67)	72 (52–90)	94 (71–120)	108 (60–134)	121 (73–148)	129 (93–154)
Deceased vs. Survivors	P = 0.185	P = 0.321	P = 0.103	P = 0.059	P = 0.106	P = 0.067	P = 0.038

mL/kg, milliliters of fluid accumulated per kg of body weight; AKI, Acute kidney injury; CI, confidence interval.

### Biphasic rate of fluid accumulation during hospitalization

**Fig 1:**
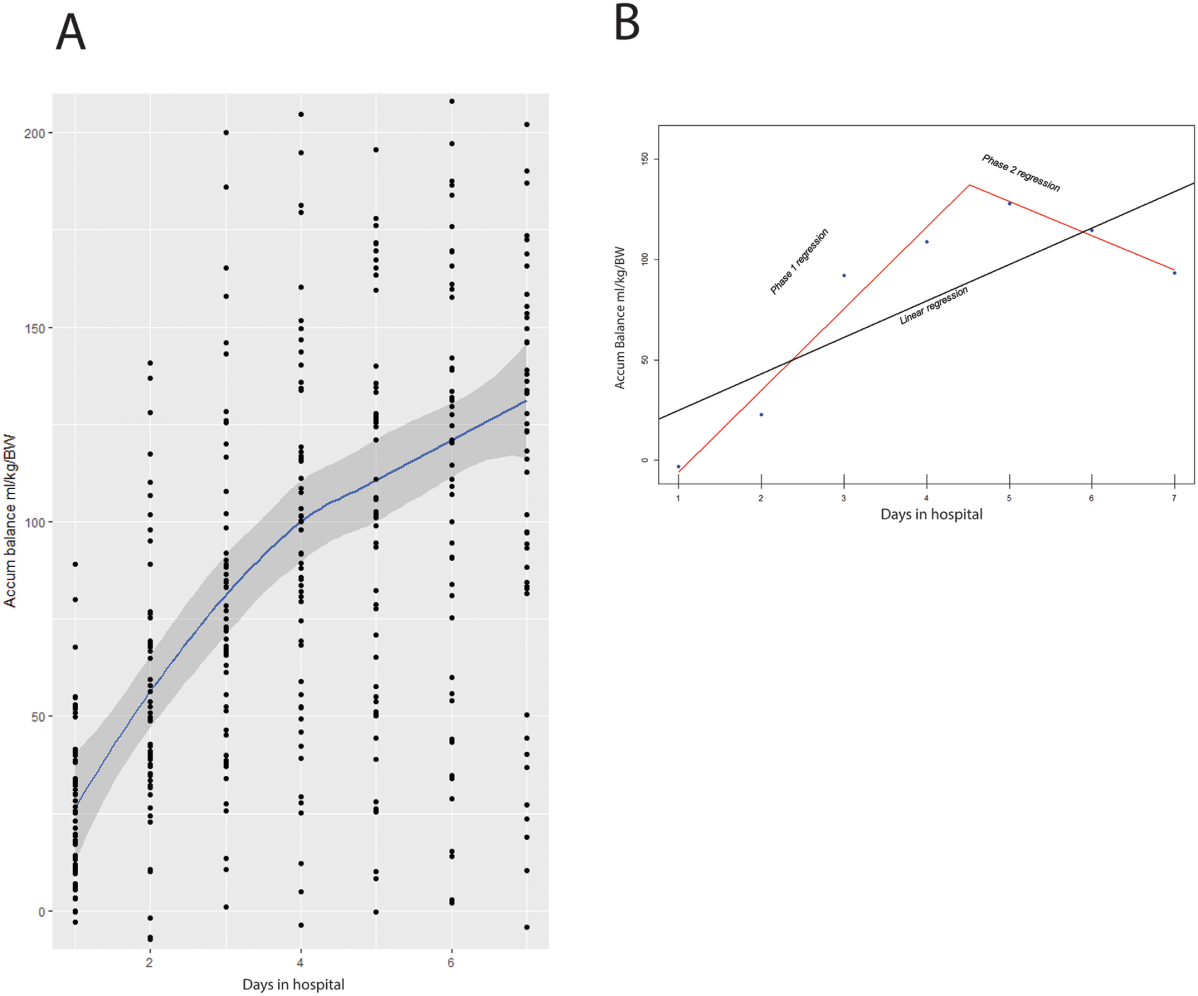
Fluid accumulation rate during hospitalization. (A) Estimated linear model including all study participants during the study period of 7 days. (B) Estimated linear model of one individual during the study period of 7 days.

Individual values of fluid accumulation of all study participants during hospitalization are shown in Fig 1A. Fluid accumulation tended to have two phases during the study period of 7 days. That is, the first phase of approximately 3 to 4 days at a high rate of fluid balance increase, followed by a subsequent phase defined by a slower rate of fluid accumulation. For each individual, we calculated the slope of the first and the second phases by using segmented linear regression. The estimated linear model of one individual is shown in Fig 1B. In this case, the Phase I of approximately 4 days at a high rate of fluid accumulation, was followed by Phase II defined by a slower rate of fluid accumulation.

### Rates of fluid accumulation in the different outcomes

**Fig 2:**
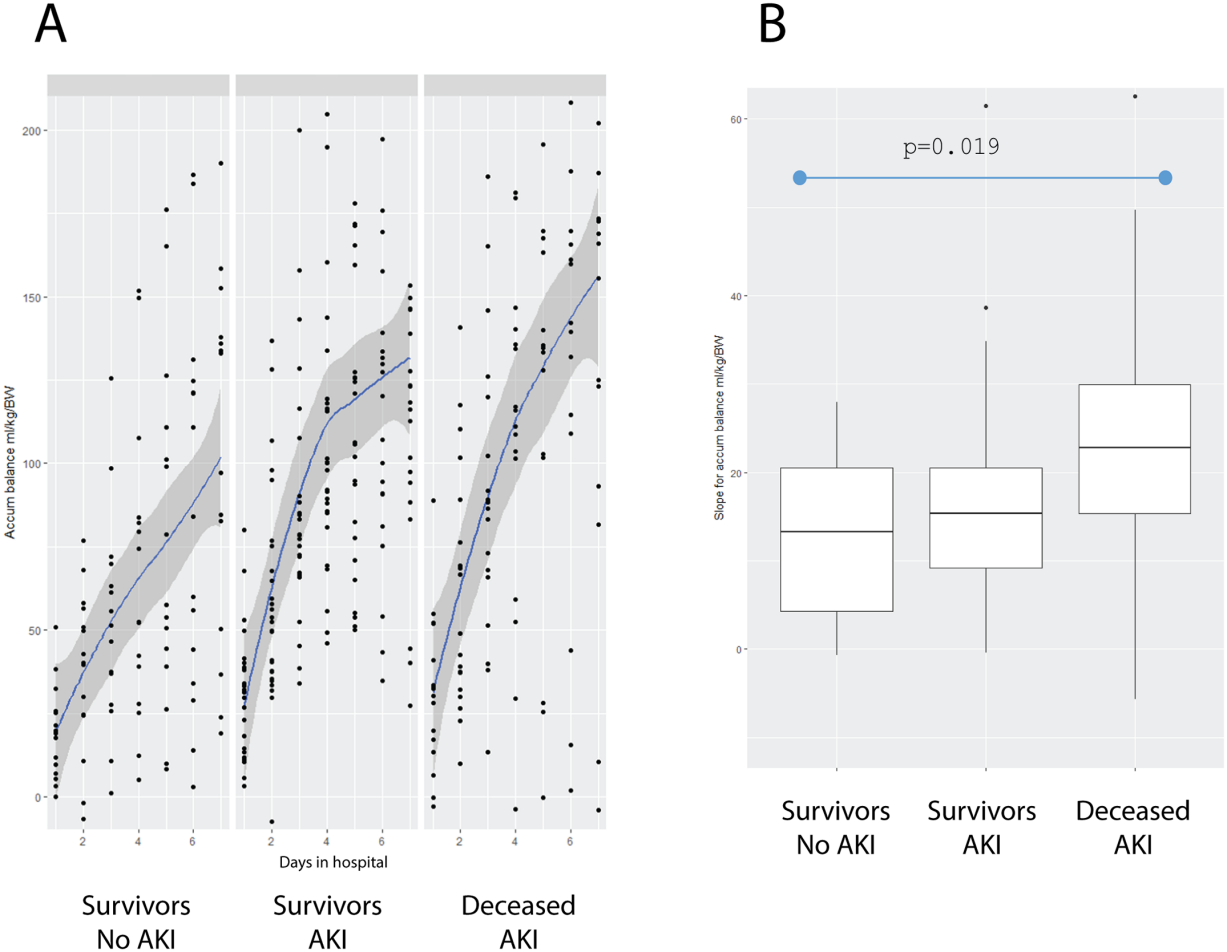
Fluid accumulation rate per groups. (A) Estimated linear model in the group of Survivors-No AKI; the Survivors-AKI Group; and the Deceased-AKI group during the study period of 7 days. (B) The different slopes in the group of Survivors-No AKI; the Survivors-AKI group; and the Deceased-AKI group during the study period of 7 days.

We explored if the rates of fluid accumulation per day were related to the different outcomes. For this, individuals who did not develop AKI and survived, constituted the Survivors-No AKI group; individuals with AKI constituted the Survivors-AKI group; and deceased individuals with AKI constituted the Deceased-AKI group. Two patients who did not develop AKI, died during the first 3 days of hospitalization. These patients were not included in this analysis, as this time period was deemed insufficient for assessing the effects of fluid accumulation. Fig 2A shows that the Survivors-No AKI group had the slower rate of fluid accumulation; the Survivors-AKI group had an intermediate rate of fluid accumulation; and the Deceased-AKI group had the highest rate of fluid accumulation, expressed as a higher slope (Survivors-No AKI group = 13.31 ml/kg/body weight vs. Deceased-AKI group = 22.76 ml/kg/body weight; p = 0.019, Fig 2B).

**Fig 3:**
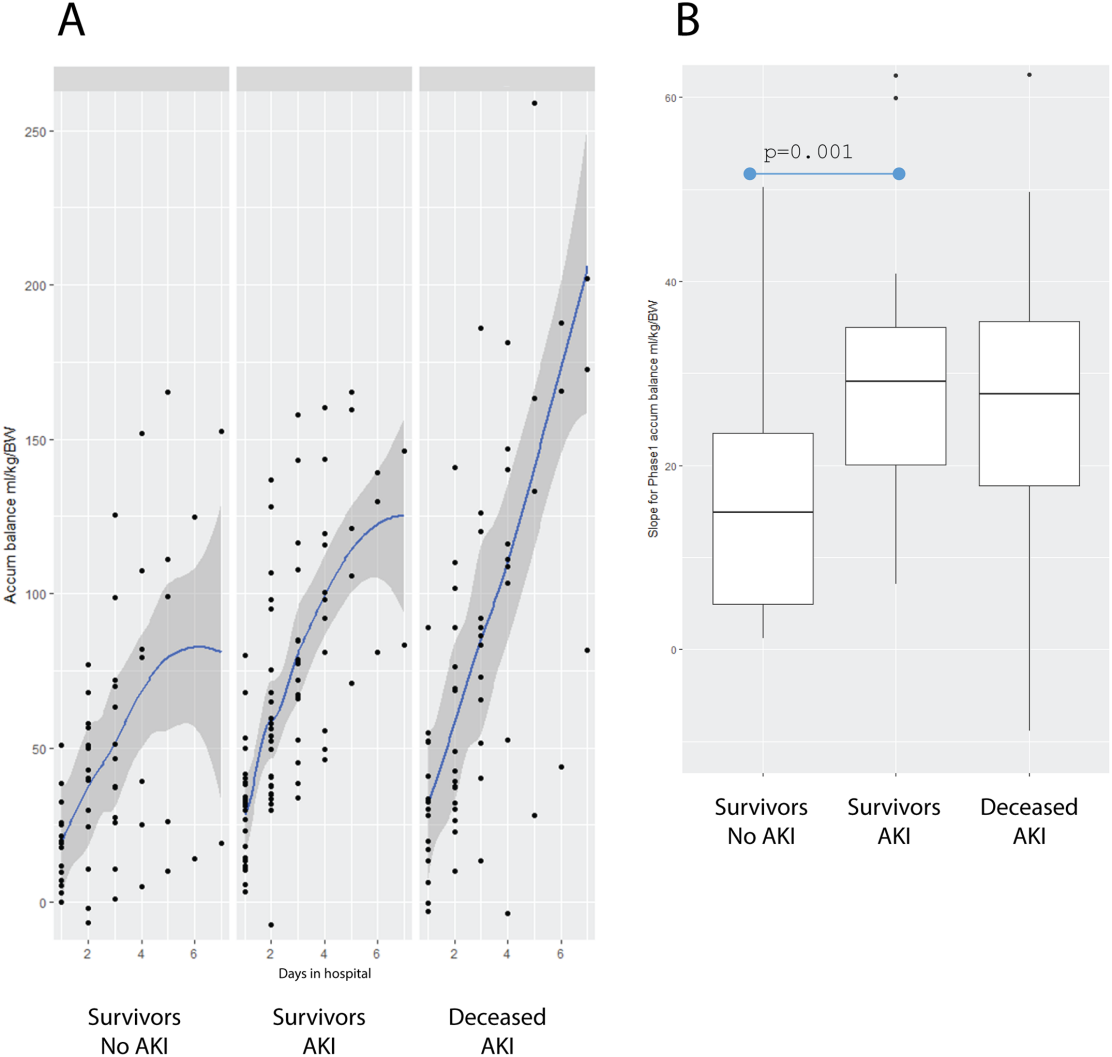
Phase I of fluid accumulation rate. (A) Estimated linear model in the group of Survivors-No AKI; the Survivors-AKI group; and the Deceased-AKI group during Phase I. (B) The different slopes in the group of Survivors-No AKI; the Survivors-AKI group; and the Deceased-AKI group during Phase I.

Considering that Phase I was defined by the highest rate of fluid accumulation in all three groups, we compared the linear segment corresponding to this initial phase among groups (Fig 3A). The Survivors-No AKI group had a significant slower rate of fluid accumulation than the Survivors-AKI group (Survivors-No AKI = 14.91 ml/kg/body weight vs. the Survivors-AKI group = 28.49 ml/kg/body weight, p = 0.001). Rates of fluid accumulation were similar in the Survivors-AKI group and the Deceased-AKI group (Fig 3B).

## Discussion

We explored the risk factors associated with the development of AKI and the relation of the accumulated fluid balance on AKI and mortality, in a homogeneous population with primary acute respiratory distress syndrome caused by influenza A H1N1 virus infection. By using the KDIGO classification, we found that 71.6% of individuals developed AKI. This proportion is similar to that found in a Canadian cohort, of 66.7% using the RIFLE classification [3]. AKI had a significant association with the use of nephrotoxic drugs during hospitalization. In agreement with a previous study in critically ill patients with lung disease, we also found a significant association between AKI and PEEP level >10 cm H_2_O on admission [16]. Male gender had a protective effect for AKI. Other factors previously associated with AKI include vasopressor use, mechanical ventilation, high APACHE II score, severe acidosis, high levels of C-reactive protein and lactic dehydrogenase upon ICU admission, longer stays in the ICU [17], high body mass index, history of asthma [3], high SOFA score, and greater incidence of shock, multiorgan dysfunction syndrome and coinfection [18]. In our cohort, the AKI group had higher PSI, APACHE II and SOFA scores on admission, as well as longer hospital stays and mechanical ventilation periods than the non-AKI group, but these differences were non-significant. We found lower PaO_2_/FiO_2_ in the AKI than in the non-AKI group, as well as lower PaO_2_/FiO_2_ in deceased individuals than in survivors, but lack of statistical significance in this parameter was probably affected by the inclusion criteria of PaO_2_/FiO_2_ <200 mm Hg on admission.

In line with previous studies, mortality was significantly more frequent in the AKI group than in the non-AKI group [16, 19, 20]. The use of nephrotoxic drugs during hospitalization and older age were significantly associated with mortality. Our findings were consistent with previous reports indicating that PSI, APACHE II and SOFA scores on admission were significantly higher in deceased patients infected with influenza A H1N1 than in survivors [18, 21].

We found a significant association between FO and AKI. Likewise, death was more frequent in the group with FO, but the limited number of deceased patients may have provided insufficient evidence to establish a difference between groups. Fluid accumulation tended to have two phases during the study period of 7 days. The first phase was defined by a high rate of fluid balance increase, followed by a subsequent phase of a slower rate of fluid accumulation. The most relevant finding of our study was that the phase of higher fluid accumulation, was significantly associated with AKI and mortality. Our results are consistent with a previous study using the concept of "slope", and showing that the velocity of fluid accumulation is important, and the more rapidly fluid accumulation occurs, the higher the risk of dying [22]. Therefore, we consider that not only the fluid overload, but also a rapid fluid balance increase, are associated with AKI and mortality. The association between fluid accumulation and adverse outcomes is well established. In ICU patients with sepsis, a positive fluid balance has been consistently identified as an independent risk factor for the incidence of AKI [23] and mortality [24–26], while a negative fluid balance is associated with survival and preservation or organ function [27–29]. Development of AKI also has important implications for utilization of health resources within and outside of the ICU. In some cases, the consequent development of chronic kidney disease may lead to renal replacement therapy [20]. In order to minimize the adverse consequences of FO, we support the approach of resuscitation in acute illness, with an early transition to neutral and then negative fluid balances [5]. The optimal fluid status should be individualized and even in the same patient, the optimal fluid status should be adjusted when the condition changes [30].

The main limitation of our study is derived from the retrospective design. That is, we found an association of fluid accumulation with AKI and mortality, but the causal mechanism of fluid accumulation and the different strategies of fluid management as therapy for AKI should be evaluated in randomized controlled trials. Another limitation of our study is that we retrieved information about fluid balance and development of AKI during hospitalization, but a longer observation period would have provided additional information regarding the clinical outcome and the impact of AKI in the population studied. Namely, we are not reporting the proportion of individuals developing chronic renal disease in the group with AKI. An additional limitation of our study is that we were unable to determine if the positive fluid balance observed in some individuals was caused by oliguria, fluid administration or a combination of both. Finally, our study was conducted in a national referral center for respiratory diseases, and this represents a potential source of referral bias. As we included previously health individuals with severe pneumonia caused by influenza A H1N, most of them had no chronic comorbidities. By consequence, reported risk factors for the development of AKI in ICU patients such as past history of chronic heart failure, lymphoma, leukemia, cirrhosis [31], peripheral vascular disease, diabetes, high blood pressure, heart failure, renal disease and hepatic disease [32] were underrepresented in our population.

## Conclusions

Our results support previous findings in critically ill patients, indicating that fluid overload and rapid fluid balance increase are associated with AKI and mortality. Scientific evidence obtained from randomized clinical trials exploring more rational and flexible approaches to fluid therapy is required for minimization of the adverse consequences of fluid overload.

## Supporting information

S1 File Raw data. This file includes the minimal anonymized data set necessary to replicate our study findings.
(CSV)
